# Protein folding: Funnel model revised

**DOI:** 10.1016/j.csbj.2024.10.030

**Published:** 2024-10-21

**Authors:** Irena Roterman, Mateusz Slupina, Leszek Konieczny

**Affiliations:** a Department of Bioinformatics and Telemedicine, Jagiellonian University Medical College, Medyczna 7, 30-688 Kraków, Poland; b Chair of Medical Biochemistry, Jagiellonian University Medical College, Kopernika 7, 31-034 Kraków, Poland

**Keywords:** Funnel model, Protein folding, External force field, Membrane proteins, Chaperone, Chaperonin, Hydrophobicity, Down-hill, Fast-folding, Enzymes, *in silico* analysis

## Abstract

The spatial structure of proteins, largely determined by their amino acid sequences, is also dependent on the environmental conditions under which the folding process takes place. In aqueous environments, exposure of polar amino acids is the driving factor, whereas protein stabilization in amphipathic membranes requires exposure to hydrophobic residues. This observation can be extended to all other environmental conditions under which proteins exhibit biological activity and, most importantly, to the folding process. The fuzzy oil drop (FOD) model assumes a centric location of hydrophobic residues (hydrophobic core) with exposure of polar residues towards the aqueous environment, as the influence of the aqueous environment is extended to include the contribution of other non-aqueous factors, enabling the assessment of their influence on protein structuring. The application of the modified FOD model (FOD-M) we have developed allows the environment to be represented as an external force field in the form of a continuum. The role of environmental conditions allows modification of the funnel model expressing the localization of the energy minimum as dependent on external conditions expressed by the K scale, where K measures the degree of other than polar water factors participating in folding process.

## Graphical Abstract

**Figure:**
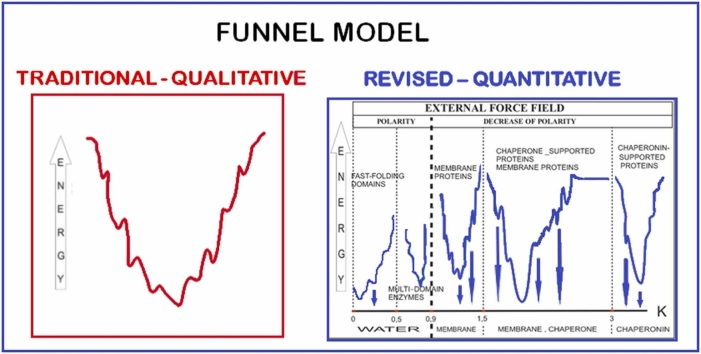

## 1 Introduction

In terms of the fundamental process of protein folding, the following question remains: Why do they fold the way they do? The general approach to predicting protein structures based on optimization of internal interactions (force field) focuses on the search for structures representing the minimum internal energy state. Combined efforts to apply the best procedures in the WeFold project have not yielded significant progress; the results are comparable to those obtained by individual teams [1]. Similar conclusions concerning the molecular mechanics of protein folding have been drawn from a diversity of interpretations [2], [3], [4].

An obvious prominent aspect of this analysis is the introduction of artificial intelligence (AI) to the AlphaFold model, which has brought significant advances to the field [5]. Protein structure prediction using available and continuously developed computer programs based on ab initio (new fold) [6] and comparative analysis [7] techniques has recently been extended to include tools based on artificial intelligence [8], [9].

The progress seen in the history of Critical Assessment of Structure Prediction (CASP), a research community collaborative [10], raises the problem of variation in the degree of correctness of structure prediction: a given tool can yield a very good result in one case and a very poor result for another protein. It can be assumed that it is the proteins themselves that vary (CASP) project distinguishes ‘easy’ and ‘hard’ proteins [11]). In the present study, we aimed to answer these questions by introducing environmental conditions that direct the folding process differently.

It is a truism that all life (mainly consisting of proteins) is inherently linked to the environment of water, the properties of which are still not fully understood (including the atypical variation in density related to temperature). In fact, only the structure of ice, that is, the solid phase of water, is known to a satisfactory degree.

An aqueous environment is a polar environment that favors interactions with polar systems. An object that is particularly ‘foreign’ to water is a hydrophobic object; thus, the mechanisms operating at the interface of phases (water/hydrophobic surface in particular) is of special interest [12], [13], [14], [15], [16], [17], [18], [19], [20], [21], [22], [23], [24].

In the fuzzy oil drop (FOD) model, water provides a specific external force field as a continuum [25]. This assumes that the folding process tends towards the maximum possible isolation of hydrophobic residues by concentrating them at the center of the protein molecule with the simultaneous exposure of polar residues. This produces entropically and enthalpically favorable contact with polar water. This phenomenon occurs when micelles composed of bipolar molecules are formed. The mixture of several bipolar molecules leads to the formation of co-micelles. The arrangement of the micelles is highly ordered, producing full surface coverage with polar groups and making the product highly soluble in aqueous environments. These structures lack specific interactions. However, the presence of proteins with a highly ordered distribution of hydrophobicity—based on the micelle-like model—as well as proteins with high solubility and no activity, is important and even critical for the life of some organisms.

The 20 amino acids can be treated as a set of 20 bipolar molecules with varying ratios of polar to hydrophobic parts. In addition, if a significant reduction in the degrees of freedom is introduced in the form of a restriction on free mobility by the present peptide bond, the formation of an optimal micelle-like arrangement is difficult in a polypeptide chain. Nevertheless, micelle-like protein structures have been identified, confirming the validity of the proposed model.

## 2 Model development

### 2.1 Expressing the role of water as a basic environment for protein activity

Micelle-like structures can be expressed by a 3D Gaussian function representing the distribution of hydrophobicity, with a maximum at the center and a polarity at the surface.

**Fig. 1:**
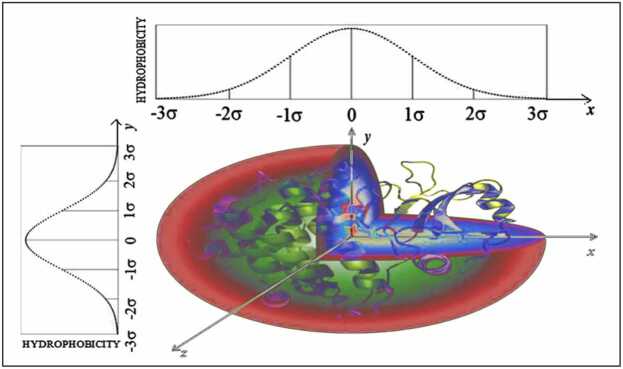
Protein molecule encapsulation in 3D Gauss function. The graphic representation of σ parameters is shown for each axis. The values of σ parameters follow the three sigma rule: the longest distance (position of particular effective atom – average position of atoms belonging to certain residue) is expressed as 3σ. The polar surface is shown here in red and the hydrophobic core in blue and green.

The protein molecule is encapsulated in an ellipsoid of a particular size and shape (Fig. 1).

The size is expressed by the Gaussian function parameter σ. In the 3D Gaussian function, the size with respect to the x, y, and z directions (axes) is defined (Fig. 1). Therefore, the value of the 3D Gaussian function encapsulating the body of the protein (proteins are predominantly globular) can determine the level of idealized hydrophobicity at any point in the protein (at the positions of effective atoms, in particular, the effective atom):

(1) $$T_{i}=\frac{1}{\sum_{i=1}^{N}T_{i}}\exp\left[\frac{-{\left(x_{i}-\bar{x}\right)}^{2}}{2\sigma_{x}^{2}}\right]\exp\left[\frac{-{\left(y_{i}-\bar{y}\right)}^{2}}{2\sigma_{y}^{2}}\right]\exp\left[\frac{-{\left(z_{i}-\bar{z}\right)}^{2}}{2\sigma_{z}^{2}}\right]$$

where *x*_*i*_*, y*_*i*_*,* and *z*_*i*_ are the coordinates that express the position of *i-*th effective atom. The size and shape of the protein are expressed by appropriately chosen values of the parameters *σ*_*X*_*, σ*_*Y*,_ and *σ*_*Z*_ (Fig. 1) where N denotes the number of amino acids in the protein.

The value of the 3D Gaussian function (*T*_*i*_) assigned to the position of the effective atom expresses the expected level, assuming an idealized micelle-like distribution. However, the actual hydrophobicity distribution may differ depending on the intrinsic hydrophobicity of each amino acid and the distance between interacting residues. This interaction can be expressed by the following model [26]:

(2) $$O_{i}=\frac{1}{\sum_{i=1}^{N}O_{i}}\sum_{i=1}^{N}\left\{ \begin{array}{l} (H_{i}^{r}+H_{j}^{r})(1-\frac{1}{2}(7{\left(\frac{r_{ij}}{c}\right)}^{2}-9{\left(\frac{r_{ij}}{c}\right)}^{4}+5{\left(\frac{r_{ij}}{c}\right)}^{6}-{\left(\frac{r_{ij}}{c}\right)}^{8})),forr_{ij}\leq c\\ 0,forr_{ij}>c \end{array}\right.$$

where r_ij_ is the distance between the positions of the effective atoms with c= 9 Ǻ (cutoff distance) and *H*^*r*^ is the intrinsic hydrophobicity (any scale can be applied). This equation represents the empirical function O, meaning that there is no explanation for the form of the function as introduced by M. Levitt [26]. To compare the distributions T and O, both shall be normalized (sum of all *T*_*i*_ and *O*_*i*_, respectively, shall be equal to 1.0). This is represented by the first factor in (1), (2). After this operation, both distributions can be compared using divergence entropy [27].

(3) $$D_{KL}(O\left|T\right)=\sum_{i=1}^{N}O_{i}\log_{2}\frac{O_{i}}{T_{i}}$$

The *D*_*KL*_ value expresses entropy that is not interpretable (the index *KL* stands from Kullback-Leibler). The introduction of a reference distribution that is opposite to the distribution with a central concentration of hydrophobicity (hydrophobic core), in the form of a uniform distribution of hydrophobicity throughout the protein, makes possible the comparable analysis. All effective atoms were assigned an equal level of hydrophobicity, *Ri= 1/N*, where *N* is the number of amino acids in the polypeptide chain.

Determining the *D*_*KL*_ values for the *O|T* and *O|R* relationships allows for an assessment of the degree of similarity of the *O* distribution to either the *T* or *R* distribution. The relationship between them can be expressed by relative distance (RD) in the following manner:

(4) $$RD=\frac{D_{KL}(O\left|T\right)}{D_{KL}(O\left|T\right)+D_{KL}(O\left|R\right)}$$

A value of *RD* < 0.5 indicates a distribution close to that of a hydrophobic core. Otherwise, the protein structure is characterized as lacking the presence of a hydrophobic core. The *RD* parameter takes real values in the interval [0,1] continuously. Thus, comparative assessment of a set of proteins from the perspective of hydrophobicity distribution within the protein body is possible.

The *RD* parameter can be determined for a single chain—as well as for a complex containing any number of chains—by spanning a 3D Gaussian function over the entire complex. It is also possible to determine the status of a selected fragment of a structure, such as a chain within a given complex (or a domain as part of a single chain). In such cases, the selected fragment described by the values *T*_*i*,_ *O*_*i*,_ and *R*_*i*_ after normalizing these values for the selected section, can be described by the value *RD* for the specific fragment. This value determines the extent to which the selected fragment participates locally in the generation of a hydrophobic core or to what extent it locally disrupts this type of ordering.

*Ti, Oi,* and *Ri* express the levels of hydrophobicity attributed to the *i*^th^ residue. The symbols *T, O*, and *R* denote the types of distributions under consideration.

The *RD* parameter can also be determined by stepwise elimination of selected amino acids; for example, amino acids showing a high divergence between *T*_*i*_ and *O*_*i*_. By eliminating such residues (e.g., in the case of *RD* > 0.5) until an *RD* < 0.5 is obtained, it is possible to identify the part of the protein that represents an ordering that matches the micelle-like arrangement. This is important, for example in determining protein solubility. Residues with a large difference between *T*_*i*_ and *O*_*i*_ can be treated as local disorder and eliminated. The next question concerns the cause and effect of this disorder. A local disturbance of micelle-like ordering carries encoded information. Indeed, residues with local hydrophobicity deficits are often catalytic residues located in the substrate-binding cavities [28]. Local excess hydrophobicity may define the site for a potential interaction leading to the formation of a complex and indicates the components of the interface. Permanent and nonpermanent complexes should be distinguished. Permanent complexes are structured as described by the model, whereas non-permanent complexes represent the ordering expressed by higher RD values for monomers as well as for the interface. This is observed for example in tubulin, the folding of which requires lower level the water influence on its structuralization [29].

The elimination of well-defined residues (the source of high RD values, RD > 0.5) lowers the RD value. This procedure allows for the identification of a part of the protein ordered according to the micelle-like organization responsible for protein solubility. Intrinsically disordered proteins identified as lacking secondary structure order appear surprisingly highly ordered based on their hydrophobic distribution [30]. In basic biochemistry, the presence of a hydrophobic core is considered a factor in tertiary structure stabilization. The stability of these proteins is dependent on their surrounding environment. Changes in the external conditions make them mobile and dynamic. It is likely that their biological activities require such specificity.

### 2.2 Expressing the role of membrane environment influencing the structuralization of membrane proteins

The cell membrane is a separate environment for protein activity. In contrast to the aqueous environment, in the cell membrane the exposure of hydrophobic residues is expected to stabilize the molecule through interaction with the hydrophobic membrane. Thus, the distribution of hydrophobicity (especially in the presence of a channel within the transmembrane protein) may be represented by a function complementary to the 3D Gaussian function in the form of *1-T*_*i*_. In practice, the following function is used:

(5) *M*_*i*_*= T*_*T* *MAX*_*– T_i_·*

**Fig. 2:**
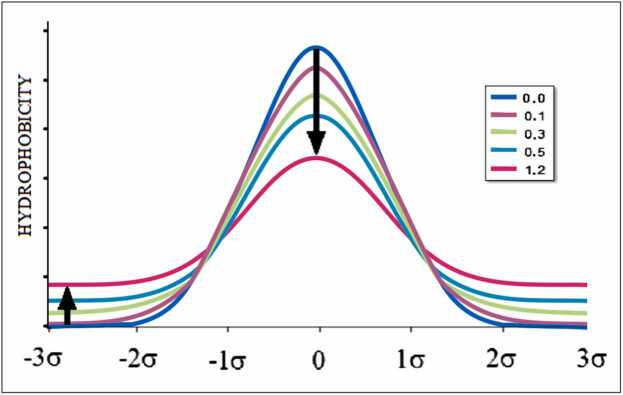
The idealized Gaussian distribution (blue) gradually changes by increased K value (as shown in legend). The hydrophobic core decreases (upper arrow) with simultaneous increase of hydrophobicity on the surface (lower arrow).

Where *T*_*MAX*_ expresses maximal value as found in T distribution. The analysis of many transmembrane proteins suggests that the distribution of hydrophobicity can be expressed as follows:

(6) Mi = Ti + [K * [T_*MAX*_*– Ti]*_*n*_*]*_*n*_

where parameter *K* determines the extent to which the 3D Gaussian function (*T*_*i*_) is modified by a function complementary to the 3D Gaussian function (Eq. 5), which represents the influence of the hydrophobicity factor. The index *n* denotes normalization. The *M* function expresses this type of modified *T* distribution, representing the effect of factors other than polar water (particularly hydrophobic factors). The *M* distribution denotes the external force field, which is reproduced by the folding process of the protein adapting to the external water conditions modified by other factors. Eq. 6 forms the basis of the FOD-M model (modified FOD model) [28]. As can be seen in Fig. 2, the Gaussian function changes gradually with an increase in K.

The degree of this influence varies. Proteins with *K* = *0* have been identified as having well-defined hydrophobic cores. Proteins with K values in the range 0 < *K* < 0.4 also turn out to describe the hydrophobicity distribution of water-soluble proteins. In their structures, a local mismatch of the micelle-like distribution (distribution according to the 3D Gaussian function system) can be identified.

**Fig. 3:**
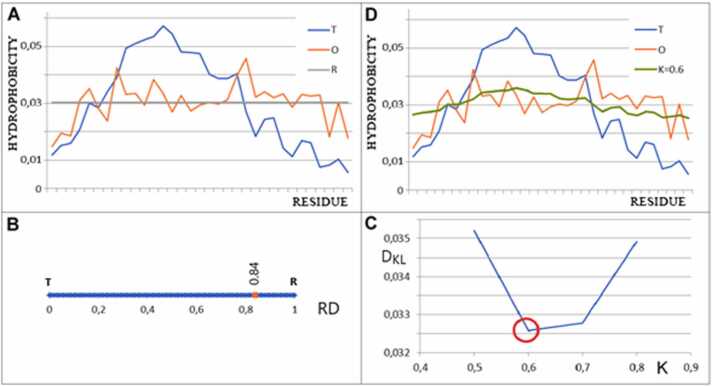
Graphical representation of the principles of the fuzzy oil drop (FOD) model and modified FOD (FOD-M). A) examples of the *T, O*, and *R* distributions. B) value of *RD* (0.84 for this example) in the absence of hydrophobic core. C) determination of the value of parameter *K* as the value for which the *D*_*KL*_ for *(O|M)* relation takes the minimum value, which is the closest approximation of the *O* distribution to the *M* distribution. D) the *T, O* and *M* distributions for *K*= 0.6.

In summary, the *RD* parameter determines the degree to which micelle-like ordering is reproduced in a given protein. The value of the *K* parameter, on the other hand, determines the extent to which an environment different from polar water contributes to the protein folding process by influencing the structure of the polypeptide chain (Fig. 3).

The relevant K value was determined as a result of the iterative procedure, as shown in Fig. 3.C. The purpose of this procedure is to determine the form of the M function with the minimal *D*_*KL*_*(O|M)* value. The *M* function represents the form of the most similar O distribution. In other words, the *O* distribution is obtained following *M* directional specificity.

## 3 Characterization of different proteins

Based on the principle of determining the values of *RD* and *K*, it is possible to present a detailed description of different proteins based on these parameters, particularly the K parameter.

### 3.1 Proteins characterized by values close to K= 0.0

*K* values close to or equal to 0 indicate proteins with structures that achieve a micelle-like distribution of hydrophobicity. Such proteins are characterized by high solubility with minimal possibility of any specific interactions. Antifreeze type II proteins belong to this group. These small globular proteins covered by a polar surface influence the ordering of water molecules, thus preventing ice-ordering in water below 0 °C. The antifreeze protein thus plays a role similar to that of salt applied during the winter season to prevent icing on the road.

Additionally, there are downhill, fast-folding, and ultra-fast-folding proteins in this group [31]. Notably, the vast majority of protein domains belong to this group. The domain treated as an individual structural unit (3D Gaussian function spanned over the domain) shows an ordering of hydrophobicity very close to a micelle-like distribution [32] (see Supplementary Materials).

**Fig. 4:**
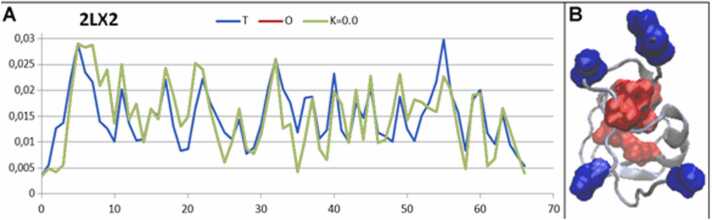
Characteristics of an antifreeze protein (PDB ID – 2LX2). A) Profiles of *T, O*, and *M* for K= 0.0 as they appear in antifreeze protein. The value of K= 0.0 causes the ideal covering of the *O* (red, nonvisible) profile by the *M* profile. On the horizontal axis are the residues in the sequence; on the vertical axis the hydrophobicity level. B) The 3D structure with hydrophobic core residues (red) and hydrophilic residues on the surface (blue), showing the localization of the residues.

This group of proteins is represented here by the antifreeze protein from *Zoarces elongatus* (PDB ID – 2LX2) [33]. The structure of this protein represents an ideal hydrophobicity distribution based on the 3D Gaussian function. This implies that the surface is covered by polar residues, which influence the ordering and structuralization of neighboring water molecules in a form adequate for charge distribution on the protein surface. Consequently, the water molecules undergo structuralization other than that occurring in ice. The source of protein structure stabilization is the well-defined hydrophobic core localized in the center of the molecule (Fig. 4).

### 3.2 Proteins in the range 0.0 < K ≤ 0.5

This group of proteins mainly comprises single-chain and single-domain enzymes. The value of *K* in this range represents the LOCAL mismatch between the *O* and *T* distributions. The *RD* value for lysozyme exceeds, at a minimum, 0.5. The elimination of three residues, including two catalytic residues, resulted in an *RD* value of < 0.5. This group also includes enzymes, such as peptidylprolyl isomerase, and others that are abundantly listed in [34]. The residues identified by introducing this local disorder are catalytic residues, allowing the identification of catalytic centers in enzymes [35].

A common feature of proteins belonging to this group is the well-defined localization of residues with a mismatch between *O*_*i*_ and *T*_*i*_ levels. A chain with an evolutionarily defined sequence cannot fully form micelle-like structures. The large proportion of micelle-like ordering—except for clearly defined locums with mismatched distributions—implies the pursuit of micelle-like structuring.

In studies of protein structure, the basic principle that the amino acid sequence in a chain determines the 3D structure can be elaborated and refined as follows: the amino acid sequence in a polypeptide chain determines how structuring is mismatched with a micelle-like form. In this way, the protein can be described as an “intelligent micelle”, due to the “intentionally” encoded presence of a local mismatch with micelle-like structuring [28] (see also Supplementary Materials).

**Fig. 5:**
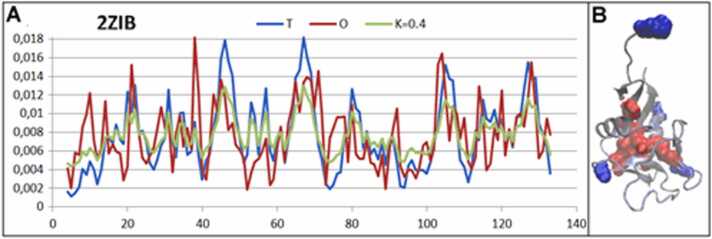
Characteristics of the antifreeze protein PDB ID–2ZIB. A) Profiles of T, O, and M for K= 0.4, revealing a micelle-like organization of hydrophobicity in the protein body. Horizontal axis: residues in the sequence; vertical axis: hydrophobicity. B) 3D structure showing the localization of hydrophobic (red) and hydrophilic (blue) residues.

Another example of this group of proteins is the antifreeze protein form *Brachyopsis rostratus* (PDB ID: 2ZIB) [36]. Here, the K value was found to be slightly higher (K=0.4); however, the molecule still represents a micelle-like organization (Fig. 5).

### 3.3 Proteins in the range 0.5 < K < 1.0

Examples of this group of proteins include structured proteins such as actin (PDB ID: 1D4X) and tubulin (PDB ID: 1FFX) [29]. Relatively high values of the *K* parameter (0.7 for actin and 0.6 for tubulins) indicate the involvement of non-aqueous factors. These two proteins represent a group in which prefoldins are involved in folding. Prefoldin is a chaperone protein that provides an external force field to prevent micelle-like structuring by isolating the folding chain from the aqueous environment. The *RD* values in the range 0.57–0.67 indicate a mismatch state dispersed along the entire chain. The absence of a well-defined hydrophobic core contributes to reduced stability and structural flexibility. Such a state of slight instability seems to make a protein more ready to interact with another protein without forming a permanent structure with it, as is the case in dystrophin, where the outcome is primarily a complex that does not allow destabilization under external stresses in which the protein functions [29], [37].

Research has shown that there are numerous enzymes in which structuring often corresponds to the range of the parameters *RD* and *K* [35] (see Supplementary Materials).

**Fig. 6:**
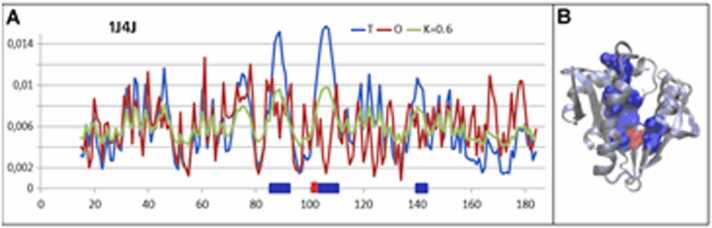
Characteristics of acetyltransferase (PDB ID 1J4J). A) The profiles *T, O*, and *M* for *K*= 0.6 allow the identification of residues causing an RD value above 0.5. The residues causing RD > 0.5, are indicated as: red, catalytic residue and blue, residues creating the cavity. The horizontal axis represents residues in the sequence and the vertical axis, hydrophobicity. B) The 3D structure shows catalytic residues (red) and cavity-generating residues (blue).

Acetyltransferase from *Pseudomonas amygdali* E.C. 2.3.1, was selected to represent the proteins characterized by K in the discussed range (PDB ID 1J4J) [38]. The structure of this protein is described by: RD= 0.592 and K= 0.6. Elimination of the catalytic residue (E103) lowers the RD value to 0.589. The catalytic residue is localized in the cavity. Ignoring the residues constructing this cavity (87–91, 103–109 and 141,142), the rest of the protein structure is characterized by a micelle-like organization (RD=0.499) (Fig. 6). This is an example of a protein that lacks micelle-like ordering; however, the source of this discordance is very well localized and limited to a few residues.

**Fig. 7:**
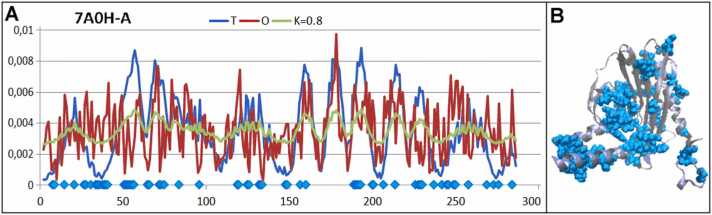
Characteristics of actin (PDB ID 7A0H-A). A) Profiles *T, O*, and *M* for K= 0.8, as found for actin. Residues colored blue represent large differences between *Ti* and *Oi*. Their positions are distributed along the whole chain (A) and in protein body (B). The horizontal axis represents residues in the sequence and the vertical axis the hydrophobicity. B) Visualization of the 3D structure of the distribution of residues—elimination of which makes the RD < 0.5. (residues distinguished as blue areas, as shown on the horizontal axis in pane A).

A protein of similar status (RD = 0.662) with K= 0.8 represents a different organization of hydrophobicity. The residues representing different statuses of *Oi* with respect to *Ti* are distributed all over the protein molecule (Fig. 7), making it impossible to identify any well-defined activity centers. Actin from *Plasmodium berghei* (PDB ID 7A0H-A) [39] is responsible for filament construction. Therefore, its structure would be expected to be less rigid to allow adaptation to different external conditions, including multicomplex construction. Therefore, the hydrophobic core of actin is not as evident as in the case of the enzyme discussed above.

The residues that were eliminated to lower the *RD* value—the blue dots on the horizontal axis in Fig. 7—showed a rather large distribution throughout the polypeptide chain without a well-defined hydrophobic core, making this molecule more elastic. The elimination of a large number of residues yielded an RD< 0.5, which suggests that the general system was not oriented toward a well-defined hydrophobic core construction.

### 3.4 Proteins with K value range of 1.0–1.5

**Fig. 8:**
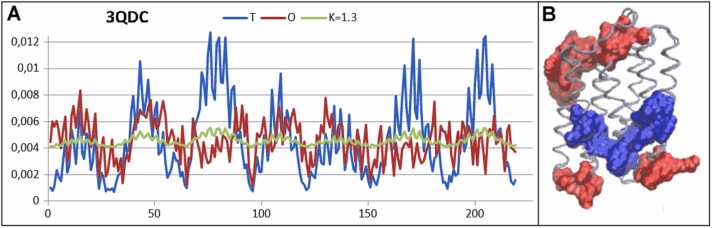
Characteristics of rhodopsin (PDB ID 3QDC). A) profiles of *T, O*, and *M* for K= 0.8, as found for rhodopsin. In A, the horizontal axis represents the residues in the sequence and the vertical axis the hydrophobicity. B) 3D structure with residues representing excess hydrophobicity (red) and hydrophobicity deficiency (blue).

Proteins in this group are mainly membrane proteins, such as rhodopsin (*K* = 1.3) as shown in Fig. 8 and in a previous study [28]. The contribution of the membrane environment is significant according to the assumptions introduced in the FOD-M model by the introduction of the *T*_*MAX*_ *– T*_*i*_ function, modifying the specificity of the aqueous environment described by the 3D Gaussian function.

Proteins in this group also include long-chain and multi-domain enzymes, including some lyases, oxygenases, and glycosidases [35]. Large proteins with a hydrophobic distribution different from that of the micelle-like arrangement are considered to provide an external force field for the active center. Catalytic reactions achieve significant modification of the energy barrier in many catalyzed processes in the presence of an appropriate force field, the specificity of which is expressed by the *K* parameter (and *RD*). By analyzing the structure of alkaline phosphatase (E.C. 3.1.3.1), which folds with the participation of a chaperone, this observation can be generalized to all proteins with a high *K* value fold with the participation and support of a chaperone (PDB ID: 6PSI) [40].

Proteins folded with the participation of a chaperone (GroEL) (PDB ID: 7LUP [41]) tend to have *K* values in this range.

A common feature of proteins whose *K* values fall within the range discussed here is the dispersed location of residues showing high variability in *O*_*i*_ versus *T*_*i*_. It is difficult to locate a specific section of a polypeptide chain with such characteristics (which is often the case for chains with a status of 0.0 <*K*<0.5).

A characteristic feature of these proteins is the profile of the *M* distribution, which takes a form similar to the *R* distribution, where there is no variation in hydrophobicity levels across the protein (or the variation is minimal). Such a state in a given protein means folding in a kind of ‘water vacuum,’ without standard water influence on the direction of the folding process. This is achieved through the involvement of chaperone proteins such as GroEL, which completely insulates the folding protein from the water environment and simultaneously introduces a specific field that acts as an external force field for the folding process (see also Supplementary Materials).

The membrane protein selected for analysis was the sensory protein rhodopsin, extracted from *Natronomonas pharaonis* (PDB ID 3QDC [42]. This protein is a helical transmembrane protein with a high K=1.3. The source of the relatively large discrepancy between the *Ti* and *Oi* levels of hydrophobicity was the presence of a channel in the central part of the molecule. The protein molecule is mainly covered by polar residues that allow transport. The surface (In contrast to water-soluble proteins) is covered with hydrophobic residues to introduce stability when in contact with the hydrophobic membrane, as illustrated in Fig. 8. The analysis of whole spectrum of different anchoring systems of membrane proteins is discussed in detail in a previous study [43].

**Fig. 9:**
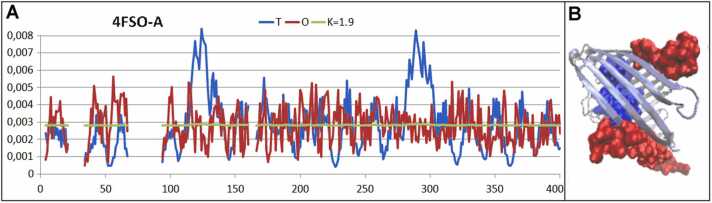
Characteristics of porin (PDB ID 4FSO). A) Profiles of T, O, and M for K= 1.9, as found for porin from *Pseudomonas aeruginosa*. The horizontal axis represents residues in the sequence and the vertical axis hydrophobicity. B) 3D structure with residues representing excess hydrophobicity (red) and hydrophobicity deficiency (blue).

Another transmembrane protein representing the beta-barrel structure selected for analysis is porin, isolated from *Pseudomonas aeruginosa* (PDB ID 4FSO) [44]. The channel of the protein is well defined by fragments of significantly lower levels of *O*_*i*_ compared with *T*_*i*_ (Fig. 9). The exposure of hydrophobic residues—enabling anchoring of the molecule in the hydrophobic membrane—is different than in previous examples, as there is a broad spectrum of anchoring systems [43].

### 3.5 Proteins in the range 1.0 < K < 4.0

Membrane proteins that act as ion channels, including the mechanosensitive channel of small conductance HpMscS and translocase, belong to this group [43].

Here, the active contribution of the hydrophobic environment of the membrane to protein structuring, especially with regard to the domains forming the transmembrane part of the complex is very strong and clear. The multi-chain structure of the transmembrane domain shows a structural form strongly dominated by a hydrophobic membrane environment.

Analysis of transmembrane proteins showed significant variation in the distribution of surface residues, exhibiting high levels of *O*_*i*_. This phenomenon can be linked to the need for mobility of the membrane protein, which, when anchored, for example, on one side only, can move to a limited degree according to the form of the anchorage [43].

**Fig. 10:**
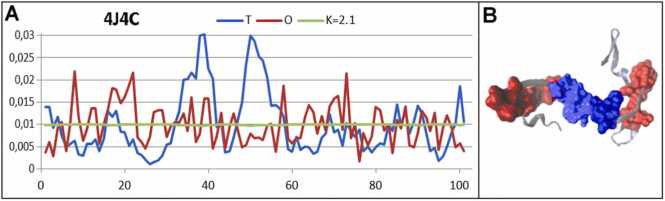
Characteristics of cyanovirin-N (PDB ID – 4J4C). A) Profiles of T, O, and M. The horizontal axis represents residues in the sequence, and the vertical axis hydrophobicity. B) 3D structure with residues representing hydrophobicity excess (red) and hydrophobicity deficiency (blue).

Among the group of proteins characterized by high K values are structuralization proteins, which can be categorized as unfolded or partially unfolded. The protein cyanovirin-N from *Nostoc ellipsosporum* (PDB ID 4J4C) [45] is an example of this class of proteins (Fig. 10).

The structure of this protein is treated as domain-swapped oligomeric suggesting “trapped” folding intermediates. Structures of this type require a “permanent chaperone” to keep their structure. This role is usually played by the second chain in domain-swapping system [46]. In other words, the individual chain cannot exist in the presented form.

### 3.6 The chaperonin structure delivering the external force field K > 4.0

The highest values of K (thus far) have been found for chaperonins delivering extremely different folding conditions with respect to the aquatic environment. The *K*= 7.0 parameter was found to describe the specificity of the external force field generated by *E. coli* co-chaperonin GroES (PDB ID - 7VWX) [41], [47] (see Supplementary Materials).

**Fig. 11:**
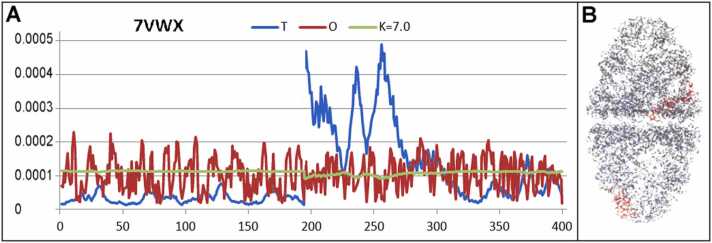
Characteristics of chaperonin GroEl:eS2. A) The T, O, and M profiles (K = 7.0). The limited fragment (first 400 aa in the PDB file) of the chains participating in the construction is presented because of the large number of residues (9146 aa). Fragments are presented in the form of profiles distinguished in B as red fragments. The horizontal axis represents residues in the sequence and the vertical axis represents hydrophobicity. B) 3D structure of GroEl:eS2 with a fragment shown in A, distinguished in red.

Analysis of *T* and *O* values (Fig. 11) shows an almost constant level for the O profile, representing either excess or deficiency of hydrophobicity in nearly all positions. The M profile takes the form of a line parallel to the x-axis. This means that the construction of the entire system generates a structure introducing a significantly different organization with respect to the conditions expected for the water environment. The interpretation of the K value in this case was quite different; it does not express the status of a molecule (complex) due to environmental influences. The GroEl:eS construction represents the environment by delivering an external force field to the proteins folded in its chambers.

Our analysis of the folding process of chains participating in this construction is focused on the status of each individual chain.

### 3.7 Predictability of structures using AlphaFold2 (AF2)

The model presented in this study is closely related to the problem of protein folding *in silico*. Predicting the 3D structure of a given amino acid sequence has been a challenge for years [48]. The CASP project, initiated in 1996, focused on monitoring progress in the discipline of protein folding simulation. The criteria for the similarity of models delivered by the participants with respect to the native structure of the protein under consideration are expressed by the GDT_TS value (the higher the value, the better the model). Many factors are taken under consideration to calculate its value – mostly focusing on geometry comparisons. The similarity can also be estimated using factors such as the hydrophobicity distribution [49].

The newly applied method based on artificial intelligence, AlphaFold2, delivers correct models [50], [51]. The FOD-M model was used to assess the accuracy of the hydrophobicity distribution in the model versus the native forms of the target molecules.

**Fig. 12:**
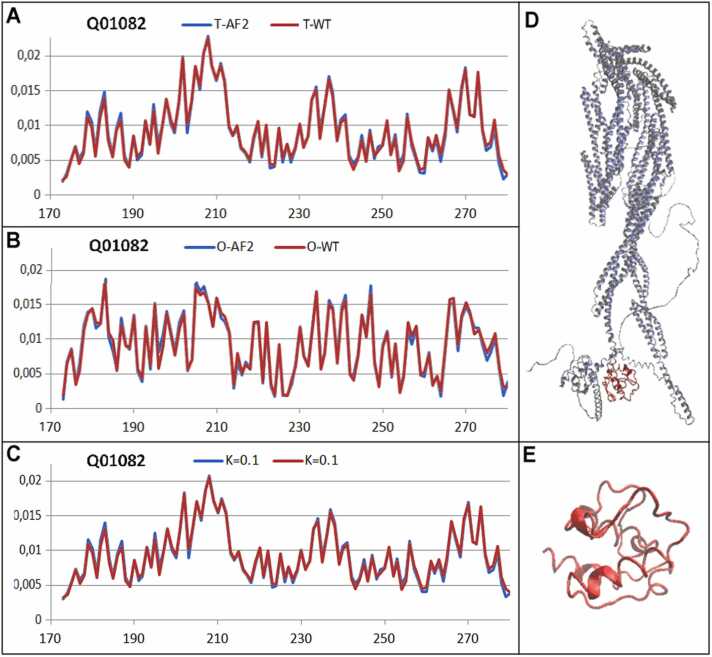
Profiles expressing the status of a domain in beta-spectrin (PDB ID 1AA2 UniProt code: Q01082): model AF2 (blue lines) and WT forms (red lines). X axis: residues in the sequence; Y axis: hydrophobicity. The profiles are: *T* (panel A), *O* (B), *M* (C). The K values are given in the legend. In A, B, and C, the horizontal axis represents the residues and the vertical axis represents the hydrophobicity. In panel D is shown the 3D structure of the complete molecule as delivered by AF2; the domain under consideration is shown in red. In panel E is shown the 3D structure of domain under comparative analysis.

**Fig. 13:**
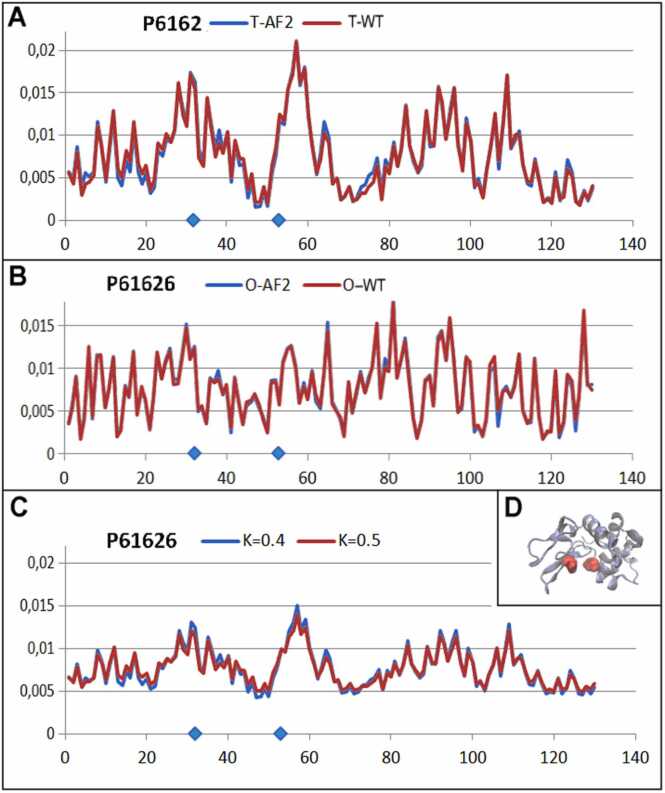
Profiles as calculated for AF2 model (blue lines) and WT form (red lines) available in PDB for tubulin gamma-1 chain (PDB ID 7SJ9 Q13509). X axis: residues in the sequence; y axis: hydrophobicity. The profiles are: *T* (panel A), *O* (B), *M* (C). The K values are given in the legends. In A, B, and C, the horizontal axis represents the residues and the vertical axis represents the hydrophobicity. D) 3D structure of protein under consideration.

**Fig. 14:**
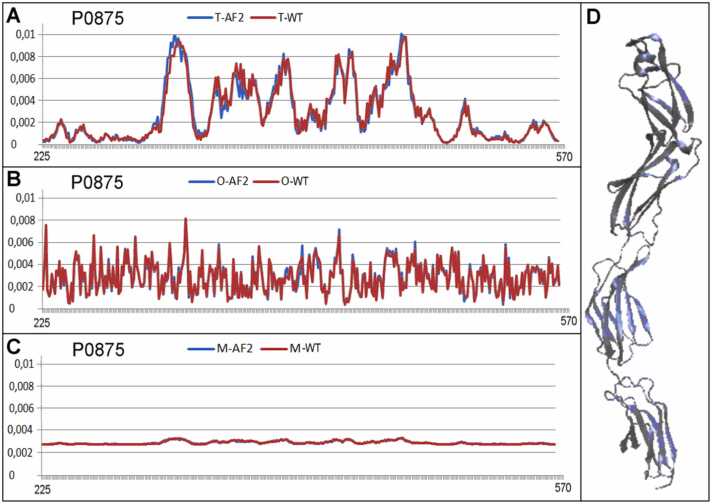
Profiles as calculated for AF2 model (blue lines) and WT form (red lines) available in PDB for human cd45 extracellular region, domains d1-d4 (PDB ID 5FMV UniProt code P08575. X axis: residues in the sequence; y axis: hydrophobicity. The profiles are: *T* (panel A), *O* (B), *M* (C). The K values are given in the legends. In A, B, and C, the horizontal axis represents the residues and the vertical axis represents the hydrophobicity. In panel D is shown the 3D structure of protein under consideration.

Three proteins representing low, medium, and high K values were selected for this comparable analysis. The selected low-K example is a domain in beta-spectrin (PDB ID 1AA2 UniProt code: Q01082 [52], [53] described by K=0.1; the medium-K example was tubulin gamma-1 chain (PDB ID 7SJ9 UniProt Q13509 [54], [55], described by K=0.6, and the high-K (K=2.2) example was the human cd45 extracellular region, domains d1-d4 (PDB ID 5FMV UniProt code P08575 [56], [57]) (Fig. 12, Fig. 13, Fig. 14).

Independent of the K value, all protein models have a high degree of concordance with the experimentally determined structures of given proteins.

Structures were generated using the server described in [58].

The structure generated by AF2 Beta-spectrin, identified by UniProt ID Q01082, delivers the structure of the complete protein. The object of analysis was limited to the domain (173–280 according to UniProt numbering). The structural prediction of this small domain in context with a very large complete molecule (2364 aa) is a challenge that can be characterized as a spectacular success. The agreement of all profiles (T, O, and M) was very high for an equal value of K= 0.1 (Fig. 12).

An example representing the structure described by K= 0.6 is the tubulin gamma-1 chain (PDB ID 7SJ9 UniProt Q13509). A comparison of the *T*, *O*, and *M* profiles is presented in Fig. 13., demonstrating the exact reconstruction of the hydrophobic distribution of the protein.

The examples analyzed show that the AF2 structure prediction is in accordance with WT structures independently of the external force field the proteins generated in native conditions.

## 4 Discussion

### 4.1 Specificity of external force field

The involvement of an external force field in the protein folding process warrants assessment of the characteristics of the field itself [28].

Analysis of protein structures emphasizes those categorized as intrinsically disordered proteins, where complex formation is largely based on matching hydrophobicity distributions, leading to structuring based on making the hydrophobic core common, irrespective of the lack of ordering in the sense of a secondary structure [28].

In contrast, the external force field in the form of a chaperone or chaperonin composed of proteins can be characterized using the *RD* and *K* parameters.

The external force field of a chaperone involved in the folding process of alkaline phosphatase (E.C. 3.1.3.1) is characterized by *K* = 1.1. The protein with its participation in folding had the same value *K* = 1.1.

**Fig. 15:**
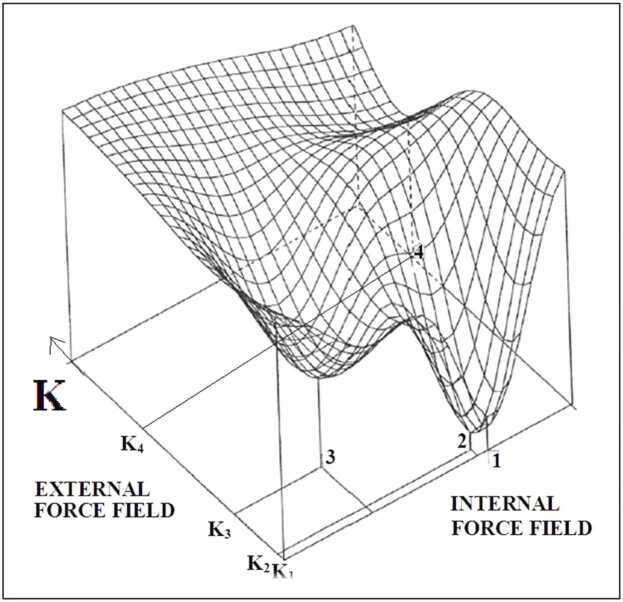
The 3D presentation of energy surface as two-variables dependent: internal force field (inter-molecular interaction) and external force field expressed by K parameter. Four minima are shown for different four K values. The internal force field is dependent on the set of inter-atomic distances [r_ij_].

Chaperonin (GroEL) supporting folding, for example, reovirus mu1/sigma3 o *K*= 1.3, demonstrates, by its structure, a value *K*= >4.0 [40]. This is an extremely large contribution from the field *T*_*MAX*_*-T*_*i*_*.* This implies that GroEL delivers a local field that is completely devoid of the influence of polar water, delivering an external force field similar to that of the *R* distribution. The force field in a protein molecule was treated as a two-variable function: internal and external (Fig. 15).

### 4.2 Protein structure prediction

The CASP project mentioned in the Introduction, in which participants use tools generated for the 3D structure prediction of proteins, reveals the dependence of the structure on the environment. The use of a single procedure (however well developed) cannot reproduce such a diverse world of proteins, with this diversity arising from the contribution of an environmental factor. Therefore, no model based on any form of ‘averaging’ the force field used in a protein prediction procedure will pass the test [59].

To conclude the presented material the summary is shown in Table 1.

**Table 1:** Variability in the spatial structure of proteins under different environmental conditions.

	K= 0.0	0.0 <K< 0.5	0.5 <K< 0.9	0.9 <K< 1.5	1.5 <K< 3.0	K> 3.0
ENVIRONMENT	WATER	WATER	CHAPERON	MEMBRANE		
AA < 100	**DOWN-HILL**	**FAST-FOLDING** **ANTIFREEZE**				
100 <AA< 200		**ENZYMES** **One domain**	**ENZYMES** **Multi-domains**			
200 <AA < 300			**ENZYMES** **Multi-domains**	**MEMBRANE** **PROTEINS**	**CHAPERONE** **MEMBRANE** **PROTEINS**	
AA > 300				**MEMBRANE** **PROTEINS**	**CHAPERONE**	**CHAPERONIN**

**Fig. 16:**
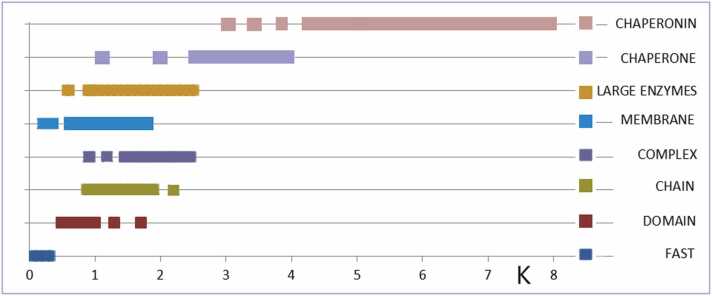
The K values as ranges characterizing the selected groups of proteins. The detailed values are given in Supplementary Materials. Category “fast” represents the fast-folding, ultra-fast-folding, down-hill and antifreeze type II. Category “chain” represents the proteins of < 200 aa chain length. Category “complex” represents the IV-order structures; “membrane” are proteins anchored in membrane including beta-barrel as well helical forms of these proteins; “large enzymes” are proteins of > 200 aa of one-domain construction, “chaperone” are supporting proteins including prefoldin; “chaperonins” are GroEL-GroES constructions. All examples are presented in details in Supplementary Materials.

A short description of the applicability of the FOD-M model is supported by a large set of results characterizing proteins using the *RD* and *K* parameters presented in the Supplementary Materials. A summary of these results is presented in Fig. 16.

The 3D structure is determined by the amino acid sequence (Anfinsen dogma [60]). It is shown that the environment in which the polypeptide chain folds influences this process. Thus two factors determine the 3D structure. The proposed model introduces quantitative participation of external factors, which together with the aa sequence produces the functional structure of the protein. As it can be seen there are no rigid limits for certain groups of proteins. Fig. 16 visualise the ranges of K values expressing the external conditions which – when applied in simulation of folding process – may deliver the proper structure. This hypothesis shall be checked by many tests.

## 5 Conclusions

The analysis revealed the importance of the external force field as a factor influencing mechanisms of protein folding and amyloid transformation in particular. This was demonstrated by analyzing the results of protein structure prediction as part of the CASP projects 13, 14, and 15, where the use of uniform force-field parameterization resulted in a variation in the correctness of the obtained models, also for programs assessed as highly reliable [49], [59].

**Fig. 17:**
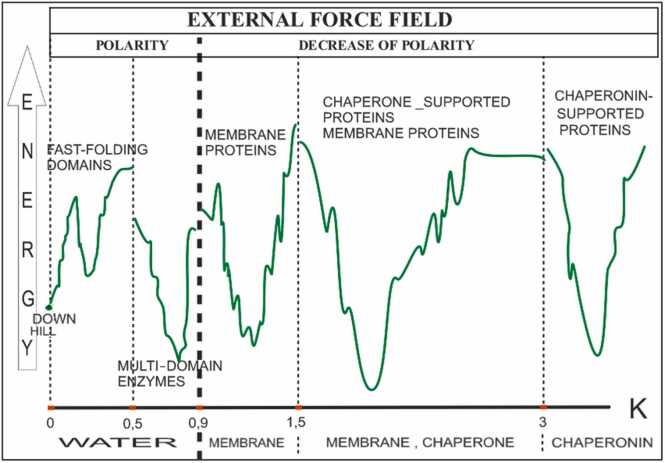
Funnel model expressing the determinants resulting from the environmental specificity expressed by the value of the K parameter.

The general conclusion allows the modification of the funnel model [61], [62], [63], [64], [65], [66], [67], [68], [69], [70] by introducing a quantitative scale on the horizontal axis (Fig. 17). This suggests that the optimal status of the protein depends on the balance between the internal and external force fields (Fig. 15).

All presented results are available (recalculation is possible) using the freely available software https://hphob.sano.science. To date, more than 300 proteins have been analyzed (Supplementary Materials). However, large-scale calculations were required.

The vertical axis in Fig. 17 indicates the energy levels that are difficult to compare in terms of the forms created in a variable environment. It cannot be ruled out that a polypeptide folded in an environment of, for example, polar water (*K*=0.0), can obtain an energy state lower than that in the native form with *K* > 0.0. However, if the goal is to obtain a biologically active form, the structure representing the energetically lower state is not important because 1) it is not possible to achieve this in a specific environment, and 2) the energetically lower state is irrelevant from the perspective of biological activity [49], [59], [71]. The overall structural stability of the protein appeared to be gradual. The construction of a well-defined hydrophobic core may introduce an almost rigid stability [37], whereas the distributed hydrophobic interaction makes elastic forms available, as observed in cytoskeletal constructions [72], [73], [74]. Construction of a hydrophobic core is critical for biological activity. Such differential stability of proteins during the folding process can be achieved under different environmental conditions. It can be expressed in the form of an external force field defined mathematically, thus allowing the simulation of the folding process *in silico*.

Significant progress in protein structure prediction introduced by the artificial intelligence approaches expressed by AlphaFold and AlphaFold2 has significant implications in pharmacology, especially in drug design. There has been significant expansion in access to the 3D structures of proteins, including applications in clinical medicine. The CASP project aims to deliver 3D structures in an accurate form to reach a solution. The model presented in this paper focuses on the search for an answer to the question: Why do proteins fold the way they do ? This question also has a practical implication: Why does the optimal force field (approach) that delivers perfect structures for one protein fail in other proteins? The answer is perhaps simple: The structures are generated according to different scenarios, depending on the environment in which they are generated. The FOD-M model attempts to express the participation of external conditions in a mathematical form, allowing its quantitative measurement.

It is suggested in conclusion to introduce the multiple criteria optimization approach:

F(r_ij_) = F [F_INT_ (r_ij_), F_EXT_(r_ij_)]

where F_INT_ (r_ij_) represents internal energy optimization and F_EXT_ (r_ij_) representing the external force field as proposed in presented FOD-M model. The final force field in proteins is the consensus of internal interactions as well as of external influence.

## Funding

Jagiellonian University, Medical College: Grant # N41/DBS/001127.

## CRediT authorship contribution statement

**Mateusz Slupina:** Software, Data curation. **Irena Roterman:** Funding acquisition, Formal analysis, Conceptualization. **Leszek Konieczny:** Supervision.

## Declaration of Competing Interest

I declare that: This The paper entitled: Protein folding: funnel model revised prepared by Irena Roterman, Mateusz Slupina, and Leszek Konieczny has not been published or presented elsewhere in part or in entirety and is not under consideration by another journal. We have read and understood your journal’s policies, and we believe that neither the manuscript nor the study violates any of these. There are no conflicts of interest to declare.

## Data Availability

The datasets used and/or analyzed in the current study are available from the corresponding author upon reasonable request.

## Footnotes

Appendix A Supplementary data associated with this article can be found in the online version at doi:10.1016/j.csbj.2024.10.030.
