# Supplementary material for: Protein folding: Funnel model revised
Source: Comput Struct Biotechnol J. 2024 Oct 21;23:3827–38. doi: 10.1016/j.csbj.2024.10.030 (PMC11550765; doi:10.1016/j.csbj.2024.10.030)
Supplement: Supplementary file 1 — Supplementary material [file mmc1.docx]

***SUPPLEMENTARY MATERIALS***

**PROTEIN FOLDING - FUNNEL MODEL REVISED**

***Roterman Irena, Konieczny Leszek***

This part of the paper (as above) presents the data necessary to support the hypothesis concerning the funnel model presentation. The horizontal axis in this model, which takes always the qualitative form, may be presented in a quantitative form. The horizontal axis in funnel model – as assumed in currently presented model based on FOD and FOD-M model – expresses the degree of external force field (external environmental conditions) participation in folding process. The data given below as well as referenced papers discuss different groups of proteins representing the structure dependent on environment (water, membrane chaperons, chaperonins). The results of analysis are presented for selected groups of proteins. The groups are defined according to increased participation of environment – according to increased K value.

***Short-chain proteins including fast-folding, down-hill, ultra-fast-folding***

Short-chain proteins represent an ordering of hydrophobicity that almost perfectly reproduces the micelle-like arrangement. These proteins are characterised by high solubility. In the case of anti-freeze type II proteins, this is critical. Their polar shell imposes an ordering of water molecules different from that in ice. By doing so, it resists freezing.

| PDP ID | CHAIN | | |
| --- | --- | --- | --- |
|  | RD | K | LENGHT  #AA |
| 2MSI  1B7I  3NLA  1KDE  2LX2  1C3Y  1W4F  1W4G  1W4H  1W4J  1W4K  2WXC  1W4E  2BTG  2BTH  5CMV  5CN4  5CN5  5CN6  5CN7  5CN8  5CNB  5CNC  5CND  5CNE  5CNF  5CNG  1A0K  1A17  1AA2  1AHQ  1A80  2LGN | 0.378  0.289  0.371  0.323  0.303  0.381  0.352  0.347  0.341  0.420  0.390  0.382  0.347  0.460  0.398  0.433  0.432  0.430  0.430  0.431  0.431  0.431  0.433  0.430  0.428  0.427  0.429  0.369  0.525  0.339  0.493  0.357  0.601 | 0.2  0.1  0.4  0.1  0.1  0.1  0.1  0.1  0.1  0.1  0.1  0.1  0.1  0.2  0.1  0.3  0.3  0.3  0.3  0.3  0.3  0.3  0.3  0.3  0.3  0.3  0.3  0.2  0.3  0.1  0.4  0.2  0.6 | 66  66  73  65  67  108  45  45  45  51  51  47  45  45  45  152  152  152  152  152  152  152  152  152  152  152  152  130  159  108  133  277  66 |

Tab. S1. The characteristics of short-chain proteins show a micelle-like ordering.

The list of proteins with very low RD and K values also includes a short-chain protein (2LGN - Lactococcin 972 - 66aa), that shows a spectacular deviation from the micelle-like arrangement. The protein is active in a periplasmic environment, and presumably this environment directs its folding in a distinct way.

The structures of additional proteins are discussed in the papers [S1-S4].

***Single-domain proteins with a length in the range of 50-200 aa***

The characteristics of single-domain proteins with relatively short chains show an ordering close to a micelle-like arrangement, although the parameter values are higher than the examples shown in Tab. S1.

This list also includes single-chain enzymes. Lysozyme (1LZ1) is an example. Such proteins are characterised by a well-defined localisation of residues showing local deviations of Oi levels from Ti levels. Elimination of these few residues from the T and O profiles results in a reduction of RD and K values to levels like the proteins in Tab. S1. Similar characteristics apply to the Beta-glycosidase (1VFF) and ribonuclease (1RAS).

| PDB ID | CHAIN | | LENGTH |
| --- | --- | --- | --- |
|  | RD | K |  |
| 1LPL  1VDN  1VH9  1PM1-X SS  2LRH  3DGP  2E3N  3VP7  4GVW  1XJU 3SS  1T0Y  1IHC  4DT5  2L6Q  2L6R  1BBL  2WXC  2P64-A  1LZ1  1RAS | 0.434  0.484  0.517  0.419  0.288  0.357  0.465  0.512  0.529  0.493  0.440  0.502  0.555  0.473  0.477  0.556  0.382  0.298  0.529  0.586 | 0.2  0.4  0.5  0.3  0.1  0.1  0.3  0.4  0.5  0.4  0.2  0.4  0.4  0.3  0.3  0.5  0.1  0.1  0.5  0.6 | 95  161  138  180  134  62  231  166  192  156  90  169  143  62  62  37  47  51  130  123 |

Tab. S2. The characteristics of single-domain proteins with a chain length in the range of 50-200 aa.

Additional proteins are discussed in the papers [S5-S9].

***Single-domain proteins with chain length of > 200 aa***

This group of 136 proteins are enzymes representing all classes of enzymes. Their general characteristics are provided in Tab. S3. Details of single-chain and single-domain enzymes were taken from available Suppl Materials for this paper [S10]

The structure generated by the long chains described by the high RD and K values suggests, one might speculate, that it could not have been formed under polar water conditions. The high RD and K values suggest an approximation of the O distribution to that of R. This is achieved with a significant contribution from non-aqueous factors (including hydrophobic factors in particular).

.

|  | RD | K | Chain length |
| --- | --- | --- | --- |
| Mean value | 0.573 | 0.64 | 353.7 |
| Min | 0.33 | 0.1 | 205 |
| Max | 0.845 | 2.7 | 2452 |
| Stand. Dev. | 0.09 | 0.35 | 301.9 |

Tab. S3. Summary characteristics of a group of single-chain, single-domain enzymes with chain length of > 200 aa.

***Single-chain, multidomain proteins***

A summary of RD and K parameter values for chains with multidomain structure shows the characteristics of the individual domains and the complete chain. The domain status is comparable to the group of proteins listed in Tab. S1 and Tab. S2. So, low RD and K parameter values. This implies the folding of single domains according to the micellization model that occurs in aqueous environments. The status of the complete chain is already expressed in terms of much higher RD and K parameter values. This means that the structural form of the chain with increased RD and K values carries the biological function encoded in the structure. The 2ALA (viral protein) is worth mentioning. The structure of this protein is dominated by disulphide bonds (8 disulphide bonds), which significantly affects the ordering within this chain, providing a final structure characterised by high RD and K values.

The protein with cell division activity (1FSZ), whose domain status is assumed to show a micelle-like ordering, is an exception to this list. Together, these domains adopt an arrangement that provides the entire chain with a highly compatible micelle-like status.

| PDB ID | DOMAIN | | | CHAIN | | |
| --- | --- | --- | --- | --- | --- | --- |
|  | FRAGMENT | RD | K | RD | K | LENGTH |
| 1N0U | (3 - 221)(329 - 344)  (222 - 328)  (345 - 485)  (486 - 561)  (570 - 721)(829 - 842)  (563 - 569)(722 - 828) | 0.529  0.548  0.447  0.378  0.518  0.635 | 0.4  0.6  0.3  0.1  0.4  1.0 | 0.760 | 1.5 | 819 |
| 3QWP | (3 - 47)(183 - 273)  (48 - 96)  (97 - 182)  (273 - 353)  (355 - 427) | 0.494  0.528  0.330  0.459  0.463 | 0.4  0.4  0.2  0.3  0.3 | 0.619 | 0.8 | 425 |
| 4HL6 | (7 - 229)(322 - 381)  (230 - 321) | 0.540  0.367 | 0.4  0.1 | 0.650 | 0.9 | 375 |
| 4AC9 | A (0 - 180)  A (181 - 273)  A (274 - 389)  A (390 - 468) | 0.429  0.314  0.525  0.384 | 0.3  0.1  0.4  0.1 | A 0.703 | 0.9 | 448 |
| 3M7U | (3 - 76)(184 - 196)  (77 - 183) | 0.438  0.597 | 0.3  0.5 | 0.600 | 0.6 | 196 |
| 3STJ | (11 - 119)  (140 - 219)  (238 - 334) | 0.436  0.511  0.412 | 0.2  0.3  0.2 | 0.673 | 0.8 | 300 |
| 1I4G | (10 - 31)(115 - 233)  (32 - 114) | 0.443  0.370 | 0.3  0.1 | 0.547 | 0.6 | 224 |
| 1LTM | (51 - 97)(171 - 235)  (111 - 170)(250 - 361) | 0.665  0.632 | 0.9  0.7 | 0.665 | 0.9 | 309 |
| 1P2E | (1 - 99)  (103 - 360)(506 - 568)  (365 - 502) | 0.454  0.533  0.588 | 0.3  0.4  0.4 | 0.673 | 0.9 | 568 |
| 2Y24 | (31 44)(321 412)  (45 320) | 0.533  0.610 | 0.4  0.7 | 0.654 | 0.8 | 383 |
| 1VL6 | (0 - 153)  (154 - 376) | 0.588  0.397 | 0.6  0.2 | 0.526 | 0.4 | 377 |
| 3GUE | (8 374)  (375 482) | 0.581  0.366 | 0.7  0.1 | 0.642 | 0.9 | 468 |
| 3IM1 | (1850 1990)  (1991 - 2048)  (2049 - 2163) | 0.540  0.335  0.509 | 0.5  0.1  0.4 | 0.658 | 0.9 | 325 |
| 2ALA 8SS | (1 - 378)  (195 - 378) | 0.801  0.423 | 1.6  0.3 | 0.779 | 1.4 | 384 |
| 1BUP | (4 67)(123 187)(361 381)  (68 122)  (188 228)(313 360) | 0.452  0.424  0.526 | 0.3  0.2  0.4 | 0.589 | 0.7 | 378 |
| 1JIJ | (3 - 223)  (224 - 320) | 0.439  0.545 | 0.4  0.5 | 0.556 | 0.6 | 319 |
| 1AOA | (121 - 238)  (239 - 375) | 0.432  0.564 | 0.3  0.5 | 0.608 | 0.7 | 247 |
| 1BA0 | (4 - 67)(123 - 187)(361 - 381)  (68 - 122)  (188 - 228)(313 - 360) | 0.452  0.429  0.527 | 0.3  0.2  0.4 | 0.592 | 0.7 | 378 |
| 1FSZ | (38 - 249)  (250 - 356) | 0.355  0.348 | 0.2  0.2 | 0.454 | 0.3 | 334 |
| 1FFX | (1 - 268)  (269 - 383)  (384 - 440) | 0.501  0.523  0.653 | 0.4  0.4  0.7 | 0.571 | 0.6 | 423 |
| 1FGQ | (9 - 167)  (168 - 267)  (268 - 356)  (357 - 485)  (493 - 837) | 0.523  0.685  0.656  0.642  0.540 | 0.5  1.1  0.9  0.7  0.8 | 0.709 | 1.1 | 820 |
| 1AV4 | (9 - 95)  (96 - 203)  (204 - 627) | 0.618  0.493  0.630 | 0.5  0.4  0.6 | 0.701 | 0.9 | 620 |

Tab. S4. The characteristics of single-chain proteins with multidomain structure. The ranges specifying the individual domains and their status expressed by the RD and K parameter values have been provided. The complete chain has been described by the same parameters. The chain length expressed in the number of amino acids has also been provided.

A summary analysis of the status of the domains is presented in a large scale [S11].

***Complexes composed of single domain chains***

The summary of the results presented in Tab. reveals the mechanism of the structure of the complexes. The individual chains often show a micelle-like status, although once the complex is formed, a structure with high RD and K parameter values is obtained, as for example in the case of 1DXK (deoxy recombinant human haemoglobin). This means providing a structure with encoded information about the type of biological activity of the final form of the complex.

| PDB ID | CHAIN | | | COMPLEX | |
| --- | --- | --- | --- | --- | --- |
|  | RD | K | LENGHT | RD | K |
| 1Z69 | A - 0.612  B - 0.611  C - 0.612  D - 0.603 | 0.8  0.8  0.8  0.8 | 327  327  327  327 | 0.774 | 1.8 |
| 1DXV | A - 0.524  B - 0.442  C - 0.523  D - 0.434 | 0.4  0.3  0.4  0.3 | 141  146  141  146 | 0.735 | 1.8 |
| 1YDG | A - 0.556  B - 0.565  C - 0.560  D - 0.561  E - 0.583  F - 0.559  G - 0.555  H - 0.559 | 0.5  0.5  0.5  0.5  0.6  0.5  0.5  0.5 | 201  201  201  201  201  201  201  201 | 0.672 | 0.9 |
| 1LRT SS | A - 0.524  B - 0.525  C - 0.525  D - 0.530 | 0.4  0.4  0.4  0.4 | 338  338  338  338 | 0.721 | 1.6 |
| 3VTZ | A - 0.445  B - 0.441  C - 0.454  D - 0.462 | 0.2  0.2  0.3  0.3 | 253  253  253  253 | 0.586 | 0.6 |
| 1FP4 | A - 0.599  B - 0.579 | 0.6  0.5 | 467  522 | 0.739 | 1.7 |
| 1GEG | A - 0.529* | 0.5 | 255 | 0.709 | 1.1 |
| 2BEC | A - 0.751  B - 0.751 | 0.8  0.8 | 181  25 | 0.530 | 0.5 |
| 1AWI | A - 0.415  B - 0.439  P - 0.598 | 0.3  0.3  0.3 | 138  138  10 | 0.739 | 1.5 |
| 4JKR | A - 0.575  B - 0.547  C - 0.739  D - 0.732  E - 0.632  F - 0.791  G - 0.607  H - 0.605  I - 0.735  J - 0.737  K - 0.637  L - 0.788 | 0.6  0.5  1.2  1.5  0.9  1.8  0.6  0.6  1.1  1.6  1.0  1.6 | 227  227  1340  1342  90  481  227  227  1340  1342  90  481 | 0.747 | 1.7 |
| 1KKL | A - 0.473  B - 0.493  C - 0.491  H - 0.408  I - 0.418  J - 0.434 | 0.3  0.3  0.3  0.2  0.2  0.2 | 167  167  167  86  86  86 | 0.735 | 1.5 |
| 3M7F | A - 0.352  B - 0.405 | 0.2  0.2 | 107  135 | 0.617 | 0.7 |
| 1LRT | A - 0.524  B - 0.525  C - 0.525  D - 0.530 | 0.4  0.4  0.4  0.4 | 336  336  336  336 | 0.721 | 1.6 |
| 1AYP 7SS | A - 0.665  B - 0.573  C - 0.611  D - 0.538  E - 0.610  F - 0.543 | 0.5  0.5  0.5  0.4  0.4  0.4 | 124  124  124  124  124  124 | 0.649 | 1.3 |
| 3RHA | A - 0.613  B - 0.646 | 0.7  0.9 | 459  459 | 0.727 | 1.2 |
| 4FGE | A - 0.357  B - 0.356 | 0.2  0.2 | 150  150 | 0.691 | 1.3 |
| 1T2W | A - 0.343  B - 0.328  C - 0.367 | 0.1  0.1  0.1 | 145  132  145 | 0.603 | 0.9 |
| 1B77 | A - 0.647  B - 0.647  C - 0.633 | 0.8  0.8  0.7 | 228  228  228 | 0.701 | 1.3 |
| 1GN1 | A - 0.382  B - 0.389  C - 0.368  D - 0.370  E - 0.385  F - 0.373  G - 0.419  H - 0.389 | 0.1  0.2  0.1  0.1  0.2  0.1  0.2  0.1 | 151  151  140  151  140  151  151  151 | 0.762 | 1.9 |
| 1IA0 | A - 0.511  B - 0.521  K - 0.510 | 0.4  0.2  0.4 | 440  427  328 | 0.656 | 0.9 |
| 1B9X | A - 0.413  B - 0.606  C - 0.640 | 0.3  0.6  0.6 | 340  68  169 | 0.584 | 0.6 |
| 1BHW | A - 0.664  B - 0.665 | 0.8  0.8 | 392  392 | 0.774 | 2.1 |
| 3KO5 | A - 0.339  B - 0.310 | 0.2  0.1 | 152  152 | 0.371 | 0.2 |

Tab. S5. The characteristics of complexes with single-domain chain structure. The status of each chain expressed in terms of the RD and K parameter values, chain length and status of the complex have been provided.

Models for the construction of complexes based on the structure of a common hydrophobic nucleus have been discussed in [S12].

***Protein complexes - multidomain chains***

The summary presented in Tab. S5. reveals the relationships between the status of single domains, chains and the complex. The vast majority of domains show a status with a distribution very close to a micelle-like arrangement. It should be noted that these domains are assessed in the form they represent within the complex. It should be assumed that a certain degree of matching to the complete arrangement of the complex occurs. The domains with a much longer chain (e.g. domain 3V0A (453 484)+(544 844)) are the exceptions. Any deviation from the proposed rule is anticipated due to the subordination of structuring to expected biological activity. Its record very often takes the form of an exception to the rule. The amount of information increases as the probability of a particular event decreases. Hence, coding a suitable arrangement to guarantee activity should involve a high level of uncertainty (predictability). Following the rules would limit the spectrum of possibilities related to biological activity.

A regularity that can be observed is increasing RD and K values when moving from domains through the chain structure with maximum RD and K values for complexes.

RD values exceeding the conventional limit for a micelle-like system (RD=0.5) at low K values indicate a condition where the cause of the elevated RD value is due to the status of the normally defined low number of residuals showing divergent Ti and Oi values. Such local inconsistency is in most cases due to the specificity of the sequence, for which it is a way of encoding specificity (irregularity carries information).

The summary of the given values reveals a mechanism for obtaining high-information systems (entropy) by means of elements (domains) with a structure compatible with a micelle-like system and therefore with a system shaped by the influence of the water environment.

| PDB ID | DOMAIN | | | CHAIN | | COMPLEX | |
| --- | --- | --- | --- | --- | --- | --- | --- |
|  | FRAGMENT | RD | K | RD | K | RD | K |
| 1Q28 | (5 - 193)  (194 - 350)  (351 - 554) | 0.410  0.363  0.490 | 0.2  0.2  0.3 | 0.650 | 0.7 | 0.742 | 1.4 |
| 3M65 | (4 - 115)  (116 - 209) | 0.455  0.509 | 0.3  0.3 | 0.620 | 0.9 | 0.735 | 2.2 |
| 3V0A | A – (1 - 433)  (453 - 484)(544 - 844)  (860 - 1090)  (1106 - 1295)  B – (1 - 502)  (503 - 833)  (834 - 1038)  (1039 - 1194) | 0.601  0.697  0.521  0.442  0.581  0.703  0.413  0.361 | 0.7  0.9  0.4  0.2  0.5  0.9  0.3  0.2 | A 0.773  B 0.721 | 1.9  1.3 | 0.746 | 1.8 |
| 1RLU | (8 - 217)  (218 - 312) | 0.380  0.522 | 0.2  0.4 | A 0.535  B 0.517 | 0.5  0.5 | 0.746 | 1.5 |
| 1SA0 | (2 - 268)  (269 - 383)  (384 - 437) | 0.507  0.535  0.598 | 0.4  0.4  0.6 | A 0.572  E 0.840 | 0.6  2.0 | 0.833 | 1.9 |
| 1T3E | (318 - 341)(498 - 655)  (342 - 365)(464 - 497)  (366 - 468)  (656 - 736) | 0.391  0.388  0.377  0.356 | 0.2  0.1  0.2  0.1 | A 0.721  B 0.698 | 1.0  0.8 | 0.712 | 1.0 |
| 1TUB | (1 - 268)  (269 - 383)  (384 - 440) | 0.467  0.546  0.620 | 0.3  0.5  0.6 | A 0.511  B 0.521 | 0.4  0.5 | 0.687 | 1.1 |
| 1JFF | (2 - 268)  (269 - 383)  (384 - 439) | 0.416  0.510  0.627 | 0.3  0.4  0.7 | A 0.549  B 0.518 | 0.4  0.5 | 0.690 | 1.1 |
| 1BP3(1SS) | (202 - 301)  (302 - 404) | 0.433  0.524 | 0.3  0.4 | A 0.579  B 0.665 | 0.6  0.8 | 0.688 | 0.9 |
| 1UMD | (2 - 196)  (197 - 324) | 0.545  0.427 | 0.5  0.3 | A 0.573  B 0.660 | 0.6  0.9 | 0.693 | 1.0 |
| 1XJW | A (1 - 133)(292 - 310)  A (134 - 291)  B (8 - 100)  B (101 - 153) | 0.451  0.455  0.438  0.364 | 0.4  0.3  0.3  0.1 | A 0.640  B 0.594 | 0.8  0.6 | 0.790 | 2.1 |

Tab. S6. The characteristics of multidomain single-chain proteins. The sections for the respective domains and their status as expressed by the RD and K parameters, the status of the chains and the complex have been provided. The lengths of the individual chains expressed by the C terminal positions of the respective domains.

Other examples of multidomain single-chain enzyme structures are discussed in [S10].

***Membrane proteins***

The specificity of membrane proteins is due to the different environment in which they show their activity. These proteins are made up of helical chains (e.g. rhodopsin), beta-structure chains (beta barrel). A summary of parameters describing proteins of this type is presented in Tab. S6. The alignment of the hydrophobicity distribution in membrane proteins 2LHF and 2JMM is of note. This ordering suggests the presence of a hydrophobic nucleus. And this is indeed what is observed in these two proteins. These proteins have been shown to be highly resistant in relation to antibiotics [S13]. The ordering with the hydrophobic nucleus present may explain this feature of these proteins.

| PDB ID | RD | K | Aa number | Secondary structure |
| --- | --- | --- | --- | --- |
| 7CP9 A  6UCU A  7VD2 B  7E4I A  8W5J A  7E4H  1A0S P  1AF6 A  1AIG L,M  1BH3  1BXW  1BY3  1BY5  1IM0  1HXT  1HYN R  1BYW  1J4N  1KD6  2LHF  2JMM | 0.686  0.700  0.680  0.724  0.648  0.725  0.678  0.701  0.763  0.713  0.741  0.757  0.750  0.719  0.628  0.570  0.463  0.414  0.447  0.472  0.472 | 1.8  1.8  1.8  1.2  1.0  1.2  1.0  1.4  1.2  1.4  0.9  1.7  1.6  1.3  0.8  0.6  0.3  0.2  0.3  0.2  0.3 | 286  307  286  439  307  307  413  421  281  289  172  695  697  262  340  293  110  249  179  178  156 | Beta barrel  Beta barrel  Beta barrel Beta barrel Beta barrel Beta barrel Beta barrel Beta barrel  Helical  Beta barrel Beta barrel Beta barrel Beta barrel Beta barrel  Beta barrel  Mainly β  Mainly β  Beta barrel  Mainly β  Beta barrel  Beta barrel |

Tab. S7. The characteristics of membrane proteins. The RD and K parameter values and chain lengths are provided, as well as the form of the secondary structure.

The degree of differentiation among membrane proteins is due to their form of membrane anchorage. In fact, it turns out to be quite diverse (as shown in Tab. S7). Incomplete coverage of the protein-membrane interface in the form of exposure of hydrophobic residues enables protein mobility within the membrane.

As it will be shown in the case of rhodopsin (Tab. S7) and MSCS proteins (Tab. S8), it seems that mobility is significantly reduced. Stabilising the position of membrane proteins that are one link in a complex sequence of processes requires less mobility from outright stability.

Rhodopsins from dissimilar organisms is a separate group of proteins. The chain lengths are comparable similarly to the high RD and K parameter values typical of the membrane environment.

| RHODPSIN | RD | K | Length | Source organism |
| --- | --- | --- | --- | --- |
| 6G7H  1H68  3AM6  6SQG  5AZD | 0.677  0.785  0.675  0.631  0.693 | 0.9  1.3  1.0  0.7  0.8 | 230  218  224  210  251 | *Halobacterium salinarum*  *Natronomonas pharaonis*  *Acetabularia acetabulum*  *Lake phycodnavirus*  *Thermus thermophilus jl 18* |

Tab. S8. The characteristics of rhodopsin structures from dissimilar organisms. Chain lengths and RD and K parameters have also been provided.

A significant group of membrane proteins are those referred to as Mechanosensitive channels consisting of a trans-membrane domain and an extended non-membrane part. The status of the membrane domain (constructed from fragments of several chains) shows a rhodopsin-like status. The similarity of these domains to rhodopsin is also due to the presence of a centrally located channel and a structure made of helical chain fragments.

| MCSC | COMPLETE  STRUCTURE | | CHAIN | | MEMBRANE  DOMAIN | |
| --- | --- | --- | --- | --- | --- | --- |
| PDB ID | RD | K | RD | K | RD | K |
| 2OAU  6VYK  6VYL  6VYM  8DDL  6PWO  6PWP  6PWN  6RLD  7OO0  4HW9  5AJI  7ONL  7DLU  7OO6 | 0.770  0.718  0.753  0.765  0.799  0.748  0.791  0.775  0.746  0.758  0.766  0.745  0.737  0.742  0.747 | 1.5  1.1  1.4  1.6  1.4  1.3  1.4  1.3  1.3  1.4  1.6  1.3  1.2  1.3  1.3 | 0.852  0.772  0.811  0.801  0.816  0.793  0.830  0.817  0.807  0.744  0.846  0.770  0.830  0.815  0.826 | 2.0  1.3  1.5  1.6  1.3  1.5  1.5  1.5  1.5  1.2  2.1  1.2  1.8  1.6  1.7 | 0.774  0.697  0.713  0.767  0.773  0.729  0.761  0.804  0.720  0.743  0.734  0.749 | 1.2  0.7  0.8  1.2  0.9  0.9  0.9  1.2  0.9  0.9  0.9  0.9 |

Tab. S9. The summary of parameters describing the representatives of mechanosensitive channels. RD and K parameter values are provided for a complete complex, a single chain as well as a membrane domain.

The very high RD and K parameter values are due to an inverse distribution of the expected micelle like type. On the one hand, the exposure of hydrophobic residues within the membrane domain, but more importantly the presence of a channel in the central part of the complex, where the FOD model expects high levels of hydrophobicity.

The membrane protein group also includes antigenic outer membrane proteins. They are characterised by a highly symmetrical pipe-like structural form (C3 symmetry - axis of rotation along the pipe). The helical structure is present in much of the chain apart from short sections forming a beta barrel in the non-membrane part of the complex. The differentiation here appears to be small (Tab. S9.).

| EFFLUX TUNNEL | COMPLEX | | CHAIN | |
| --- | --- | --- | --- | --- |
|  | RD | K | RD | K |
| 5BUN  1EK9  5AZS  4Y1K | 0.809  0.805  0.782  0.807 | 1.8  1.7  1.4  1.5 | 0.827  0.835  0.808  0.849 | 1.5  1.6  1.3  1.6 |

Tab. S10. The characteristics of antigenic outer membrane proteins. The status of the complex and the single chain has been provided.

Numerous examples of membrane proteins are discussed in [S14-S19].

***Proteins providing altered environmental conditions for folding proteins***

This group of proteins includes chaperones [S20, S21] and chaperonins [S22]. They provide an environment distinct from water in which the folding process takes place. Chaperones provide an altered field by locally interacting with specific fragments of the folding chain. Prefoldin (6RN8), which is not directly involved in the folding itself, but by transporting a protein that is only partially folded into the GroEL GroES, preventing it from adopting the structure resulting from the environmental conditions of polar water also belongs to this group [S20]. GroEL GroES proteins form a specific capsule, inside which the folding process takes place in accordance with the immediate environment, which dictates the conditions that direct this process.

| PDB ID | RD | K | LENGTH |
| --- | --- | --- | --- |
| GroEL GroES | | | |
| 1SVT  1PF9  5W74  7VWX  1PCQ  8S32  1OEL  3ZQ0  1SS8  4PKO  4PKN  5OPX  1SX4  3WVL  1AON  2CGT | 0.773  0.781  0.766  0.811  0.780  0.773  0.780  0.778  0.779  0.795  0.794  0.782  0.775  0.787  0.798  0.781 | 3.3  3.6  3.8  7.0  3.4  2.9  3.0  3.8  3.1  5.2  5.1  4.8  3.5  4.8  4.1  3.7 | 524 x 14 97x7  524 x 14 97 x 7  477 x 8  524 x 14 97 x 14  524 x 14 97 x 14  524 x 14 97 x 14  524 x 7  524 x 14 97 x 7  524 x 7  524 x 16 95 x 16  52 x 14 92 x 14  520 x 14 92 x 14  524 x 14 97 x 14  524 x 16 97 x 16  524x14 97x7  524x14 97x7 |
| Mm cpn | | | |
| 3IYF  3IZH  3IZI  3IZJ  3IZK  3IZM  3IZN  3LOS | 0.810  0.809  0.779  0.777  0.794  0.780  0.774  0.798 | 3.2  4.6  5.5  5.1  8.0  5.4  6.3  4.2 | 491 x 16  513 x 16  513 x 16  513 x 16  513 x 16  513 x 16  513 x 16  532 x 16 |
| CPN RLS | | | |
| 3RUS  3RUV  3RUW | 0.744  0.739  0.738 | 2.0  1.9  1.9 | 516 x 4  516 x 4  516 x 4 |
| CHAPERONE | | | |
| 1Q3S  1Q3Q  1Q3R  4XCI  1FXK  3AEI  6NR8  1QSD | 0.737  0.794  0.793  0.713  0.640  0.737  0.622  A 0.603 | 3.9  2.0  2.0  1.3  0.9  1.3  0.7  0.7 | 517 x 8  518 x 4  518 x 4  A – 449, B - 313  347  188  677  102 x 2 |

Tab. S11. Proteins accompanying the folding process. The RD and K parameter values and the size of the chains and also the complexes have been provided.

A detailed analysis of the role of prefoldin, chaperone and chaperonin is discussed in [S20-S22].

Programs used: The calculation of RD and K parameters was performer using the freely available program : <https://hphob.sano.science/> prepared by Piotr Nowakowski, Krzysztof Gądek – https://Sano Science.

**CONCLUSIONS**

The data summarised in this analysis provide information addressed to participants in the CASP project [S23, S24]. The procedures used to predict protein structures, including single domains or individual chains, should take into account a record of the environmental conditions under which the folding process is taking place. The interpretation of the K parameter suggests the involvement of a non-polar water environment including hydrophobic environment in particular. The description of the field provided by GroEl GroES allows the orientation of the folding process to be taken into account, assuming that the chain structure is adapted to the environmental conditions. Similarly, the influence of the membrane environment results in a hydrophobicity distribution that differs significantly from that of the micelle-like system.

**Acknowledgments:** The authors wish to thank to Anna Śmietańska and Zdzisław Wiśniowski for technical support. This research was carried out within the project of MSHE "Support for the activity of Centers of Excellence established in Poland under Horizon 2020" on the basis of contract number MEiN/2023/DIR/3796. This project has received funding from the EU's H2020 research and innovation programme under grant agreement No 857533. This publication is supported by Sano project, carried out within the International Research Agendas programme of FNP, co-financed by the EU under the European Regional Development Fund. Many thanks to Piotr Nowakowski and Krzysztof Gądek for preparation of the freely available program <https://hphob.sano.science>**.**

The exact RD and K parameter values for the proteins discussed here in native as well as amyloid form are available in the [S25].

**References:**

1. Roterman I, Banach M, Konieczny L. [Antifreeze proteins.](https://pubmed.ncbi.nlm.nih.gov/29379256/) Bioinformation. 2017; 13(12): 400 401. doi: 10.6026/97320630013400. eCollection 2017
2. Banach M, Stapor K, Konieczny L, Fabian P, Roterman I. [Downhill, Ultrafast and Fast Folding Proteins Revised.](https://pubmed.ncbi.nlm.nih.gov/33076540/) Int J Mol Sci. 2020; 21(20):7632. doi: 10.3390/ijms21207632
3. Banach M, Konieczny L, Roterman I. [Why do antifreeze proteins require a solenoid?](https://pubmed.ncbi.nlm.nih.gov/29054801/) Biochimie. 2018; 144:74 84. doi: 10.1016/j.biochi.2017.10.011.
4. Gadzała M, Dułak D, Kalinowska B, Baster Z, Bryliński M, Konieczny L, Banach M, Roterman I. [The aqueous environment as an active participant in the protein folding process.](https://pubmed.ncbi.nlm.nih.gov/30580160/) J Mol Graph Model. 2019; 87:227 239. doi: 10.1016/j.jmgm.2018.12.008.
5. Roterman I, Stapor K, Gądek K, Gubała T, Nowakowski P, Fabian P, Konieczny L. [New insights on the catalytic center of proteins from peptidylprolyl isomerase group based on the FOD M model.](https://pubmed.ncbi.nlm.nih.gov/37139783/) Membranes (Basel). 2021 Dec 30;12(1):50. doi: 10.3390/membranes12010050.
6. Banach M, Konieczny L, Roterman I. [Secondary and Supersecondary Structure of Proteins in Light of the Structure of Hydrophobic Cores.](https://pubmed.ncbi.nlm.nih.gov/30945229/) Methods Mol Biol. 2019; 1958:347 378. doi: 10.1007/978 1 4939 9161 7_19
7. Roterman I, Konieczny L. Protein is an intelligent micelle. Entropy (Basel). 2023; 25(6):850. doi: 10.3390/e25060850
8. Kalinowska B, Banach M, Wiśniowski Z, Konieczny L, Roterman I. [Is the hydrophobic core a universal structural element in proteins?](https://pubmed.ncbi.nlm.nih.gov/28623601/) J Mol Model. 2017 Jul;23(7):205. doi: 10.1007/s00894-017-3367-z.
9. Kalinowska B, Banach M, Konieczny L, Marchewka D, Roterman I. [Intrinsically disordered proteins--relation to general model expressing the active role of the water environment.](https://pubmed.ncbi.nlm.nih.gov/24629190/) Adv Protein Chem Struct Biol. 2014;94:315-46. doi: 10.1016/B978-0-12-800168-4.00008-1.
10. Roterman I, Konieczny L, Stapor K, Słupina M [Hydrophobicity-Based Force Field In Enzymes.](https://pubmed.ncbi.nlm.nih.gov/38405467/) ACS Omega. 2024; 9(7):8188-8203. doi: 10.1021/acsomega.3c08728. eCollection 2024 Feb 20
11. Sałapa K, Kalinowska B, Jadczyk T, Roterman I. Measurement of Hydrophobicity Distribution in Proteins - Non-redundant Protein Data Bank. Bio-Algorithms and Med.-Systems2012, 8, 327-338. <https://doi.org/10.2478/bams-2012-0023>
12. Dygut J, Kalinowska B, Banach M, Piwowar M, Konieczny L, Roterman I. [Structural Interface Forms and Their Involvement in Stabilization of Multidomain Proteins or Protein Complexes.](https://pubmed.ncbi.nlm.nih.gov/27763556/) Int J Mol Sci. 2016; 17(10):1741. doi: 10.3390/ijms17101741
13. Edrington, T.C.; Kintz, E.; Goldberg, J.B.; Tamm, L.K. Structural Basis for the Interaction of Lipopolysaccharide with Outer Membrane Protein H (OprH) from Pseudomonas aeruginosa. *J. Biol. Chem.* 2011, *286*, 39211–39223.
14. Roterman I, Stapor K, Fabian P, Konieczny L, Banach M. [Model of Environmental Membrane Field for Transmembrane Proteins.](https://pubmed.ncbi.nlm.nih.gov/33807215/) Int J Mol Sci. 2021; 22(7):3619. doi: 10.3390/ijms22073619
15. Roterman I, Stapor K, Konieczny L. [Transmembrane proteins Different anchoring systems.](https://pubmed.ncbi.nlm.nih.gov/38062872/) Proteins. 2023. doi: 10.1002/prot.26646.
16. Roterman I, Stapor K, Fabian P, Konieczny L. [Connexins and Pannexins Similarities and Differences According to the FOD M Model.](https://pubmed.ncbi.nlm.nih.gov/35884807/) Biomedicines. 2022; 10(7):1504. doi: 10.3390/biomedicines10071504
17. Roterman I, Stapor K, Konieczny L. [The Contribution of Hydrophobic Interactions to Conformational Changes of Inward/Outward Transmembrane Transport Proteins.](https://pubmed.ncbi.nlm.nih.gov/36557119/) Membranes (Basel). 2022; 12(12):1212. doi: 10.3390/membranes12121212
18. Roterman I, Stapor K, Konieczny L. [Dependence of Protein Structure on Environment: FOD Model Applied to Membrane Proteins.](https://pubmed.ncbi.nlm.nih.gov/35054576/) .J Cell Biochem. 2023; 124(6):818 835. doi: 10.1002/jcb.30407.
19. Roterman I, Stapor K, Fabian P, Konieczny L. [The Functional Significance of Hydrophobic Residue Distribution in Bacterial Beta Barrel Transmembrane Proteins.](https://pubmed.ncbi.nlm.nih.gov/34436343/) Membranes (Basel). 2021; 11(8):580. doi: 10.3390/membranes11080580
20. Roterman I, Stapor K, Konieczny L. Protein folding process – role of prefoldin. Frontiers in Chemical Sciences – 2024 – in press
21. Roterman I, Stapor K, Konieczny L. [Ab initio protein structure prediction: the necessary presence of external force field as it is delivered by Hsp40 chaperone.](https://pubmed.ncbi.nlm.nih.gov/37932669/) BMC Bioinformatics. 2023 ; 24(1):418. doi: 10.1186/s12859 023 05545 0
22. Roterman I, Stapor K, Dułak D, Konieczny L. External force field for protein folding in chaperonines – potential application in In Silico protein folding. ACS Omega – 2024, in press
23. Roterman I, Stapor K, Konieczny L. [Role of environmental specificity in CASP results.](https://pubmed.ncbi.nlm.nih.gov/37950210/) BMC Bioinformatics. 2023; 24(1):425. doi: 10.1186/s12859 023 05559 8
24. Roterman I, Sieradzan A, Stapor K, Fabian P, Wesołowski P, Konieczny L. [On the need to introduce environmental characteristics in ab initio protein structure prediction using a coarse grained UNRES force field.](https://pubmed.ncbi.nlm.nih.gov/35325843/) J Mol Graph Model. 2022; 114:108166. doi: 10.1016/j.jmgm.2022.108166.
